# STAT3 modulates CD4 ^+^ T mitochondrial dynamics and function in aging

**DOI:** 10.1111/acel.13996

**Published:** 2023-10-13

**Authors:** Emelia Zukowski, Marco Sannella, Jack Donato Rockhold, Gabriella H. Kalantar, Jingting Yu, Sara SantaCruz‐Calvo, Madison K. Kuhn, Nasun Hah, Ling Ouyang, Tzu‐Wen Wang, Lyanne Murphy, Heather Marszalkowski, Kaleigh Gibney, Micah J. Drummond, Elizabeth A. Proctor, Hatice Hasturk, Barbara S. Nikolajczyk, Leena P. Bharath

**Affiliations:** ^1^ Department of Nutrition and Public Health Merrimack College North Andover Massachusetts USA; ^2^ Department of Microbiology, Immunology and Molecular Genetics University of Kentucky Lexington Kentucky USA; ^3^ Razavi Newman Integrative Genomics and Bioinformatics Core The Salk Institute for Biological Studies La Jolla California USA; ^4^ Departments of Pharmacology and Nutritional Sciences University of Kentucky Lexington Kentucky USA; ^5^ Barnstable Brown Diabetes and Obesity Center University of Kentucky Lexington Kentucky USA; ^6^ Departments of Neurosurgery, Pharmacology, and Biomedical Engineering and Center for Neural Engineering Pennsylvania State University Hershey Pennsylvania USA; ^7^ Next Generation Sequencing Core The Salk Institute for Biological Studies La Jolla California USA; ^8^ Department of Biology Merrimack College North Andover Massachusetts USA; ^9^ Department of Physical Therapy and Athletic Training University of Utah Salt Lake City Utah USA; ^10^ Department of Engineering Science & Mechanics Pennsylvania State University Hershey Pennsylvania USA; ^11^ Forsyth Institute Cambridge Massachusetts USA

**Keywords:** aging, CD4^+^ T cells, cytokines, inflammaging, mitochondria, mitochondrial STAT3, naïve CD4^+^ T cells, Th17 cytokines

## Abstract

Aging promotes numerous intracellular changes in T cells that impact their effector function. Our data show that aging promotes an increase in the localization of STAT3 to the mitochondria (mitoSTAT3), which promotes changes in mitochondrial dynamics and function and T‐cell cytokine production. Mechanistically, mitoSTAT3 increased the activity of aging T‐cell mitochondria by increasing complex II. Limiting mitoSTAT3 using a mitochondria‐targeted STAT3 inhibitor, Mtcur‐1 lowered complex II activity, prevented age‐induced changes in mitochondrial dynamics and function, and reduced Th17 inflammation. Exogenous expression of a constitutively phosphorylated form of STAT3 in T cells from young adults mimicked changes in mitochondrial dynamics and function in T cells from older adults and partially recapitulated aging‐related cytokine profiles. Our data show the mechanistic link among mitoSTAT3, mitochondrial dynamics, function, and T‐cell cytokine production.

## Graphical abstract

Higher amounts of STAT3 localizes in the mitochondria of CD4^+^ T cells from older adults. In vitro pharmacological blockade of mitochondrial STAT3 (MitoSTAT3) prevents aging‐induced increased mitochondrial branching, along with reducing complex II activity and limiting Th17 proinflammatory cytokine production.

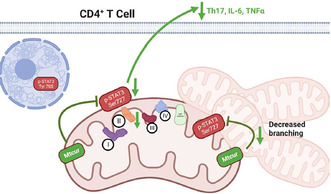

## 1 INTRODUCTION

Immune cell mitochondria are important regulators of overall health of the cell. Apart from providing energy to the cell, mitochondria regulate many functions of the immune cells including activation (Quintana et al., 2007). Mitochondria are required for antigen‐specific T cell activation by inducing reactive oxygen species signaling, differentiation (Bailis et al., 2019; Sena et al., 2013; Zhai et al., 2021), proliferation (Lepez et al., 2020) and senescence (Callender et al., 2020; Ron‐harel et al., 2015). Failure of mitochondrial homeostasis (Nowicka et al., 2021; Pandarpurkar et al., 2003), electron chain activity (Zhang et al., 2022), mitochondrial turnover (Bektas et al., 2019; Marchingo & Cantrell, 2022) or biogenesis (Akkaya et al., 2018) induce adverse structural and functional changes in the mitochondria, which can impair immune cell function. Thus, mitochondrial health has a significant role in regulating immune cell function.

CD4^+^ T cells, also known as the helper T cells, recognize cognate antigens, and then proliferate to initiate differentiation programs resulting in specific effector subsets. The cytokines produced by the subtypes of the effector T cells orchestrate the overall immune response. Numerous intracellular changes occur in T cells upon antigen recognition and activation, and include changes in metabolic programs to meet the energy demands of proliferation and cytokine production. Dysregulation of metabolic programs result in the failure of the cells to effectively perform these effector functions (Bailis et al., 2019).

Signal transducer and Activator of Transcription 3 (STAT3), a member of the STAT protein family, is phosphorylated in response to growth factors and cytokines, then translocate to the nucleus to orchestrate gene expression. The function of STAT3 as a transcription factor has been studied extensively over the past decades, but the presence of STAT3 in the mitochondria and its role in orchestrating mitochondrial function and cellular energy status has been more recently recognized. STAT3 in the mitochondria (mitoSTAT3) binds mitochondrial DNA and may be involved in direct transcriptional regulation of the mitochondrial genome (Carbognin et al., 2016). Furthermore, seminal work on mitoSTAT3 show its importance in maintaining mitochondrial respiratory chain activity (Wegrzyn et al., 2009). mitoSTAT3 also enhanced mitochondrial calcium, which strengthened interleukin‐6 (IL‐6) mediated mechanics (fitness and motility) of mouse and human CD4^+^ T cells (Valença‐Pereira et al., 2021).

We evaluated the role of mitoSTAT3 in regulating CD4^+^ T cell function during aging. We specifically evaluated the structural and functional changes in the mitochondria that are promoted by mitoSTAT3 and link between mitoSTAT3 and T‐cell cytokine production. Our data show higher mitoSTAT3 in T cells from older (O) adults compared to younger (Y) adults. Mitochondria in T cells of O adults have a complex reticular and branched structure, higher oxidative phosphorylation, and the cells produce higher quantities of proinflammatory Th17 cytokines, all of which were prevented by the pharmacological blockade of mitoSTAT3. Overexpression of STAT3 constitutively phosphorylated at serine residue 727, a modification that regulates mitochondrial localization, increased mitochondrial respiration in T cells from Y adults, and induced cytokines characteristic of Th17 inflammation, thus showing mechanistic links among STAT3, mitochondria and T cell effector function.

## 2 MATERIALS AND METHODS

### 2.1 Human adults sample collection

In accordance with the Declaration of Helsinki, informed consent for all human participants was obtained following Institutional Review Board‐approved protocols at the Forsyth Institute. Peripheral blood was obtained from normoglycemic young adults who were lean (Y; range: 25–40 y old; BMI <25 kg/m^2^) or normoglycemic older adults who were lean (O; range: 60–80 years old; BMI <25 kg/m^2^). Metabolic health was assessed by HbA1C measured by a standard commercial laboratory test (Quest Diagnostics). Subject characteristics are shown in Table 1. Exclusion criteria were smoking within the past 12 months, recent use of antibiotics or anti‐inflammatory medications that is, NSAIDs/steroids, colds/flu/COVID‐19 within the past 2 weeks, BMI > 25 kg/m^2^, type 2 diabetes or diabetes medications, and any history of cancer, hyperglycemia, and autoimmune diseases.

**TABLE 1:** Description of research subjects.

	Young	Old
Total N	11	12
Age, years [Mean (range)]*	31.81 (27–39)	68.11 (61–78)
BMI, kg m‐^2^[Mean (range)]	22.20 (19.70–23.20)	23.46 (21.10–24.00)
Females [*N* (%)]	6 (55.54%)	7 (58.33%)
Males [*N* (%)]	5(45.45%)	5 (41.66%)

#### 2.1.1 Isolation of PBMCs and T cells

Peripheral blood mononuclear cells and CD4^+^ T cells were isolated according to our established protocol as described (Bharath et al., 2020; Conway et al., 2022). Briefly, 50 mL of peripheral blood was collected into acid/citrate/dextrose containing tubes by venous puncture. PBMCs were purified by histopaque. CD4^+^ T cells were isolated from the PBMCs by negative selection using MACS columns (Miltenyi Biotech). Isolated cells were placed in −80°C for 24 h and then stored at −190°C in liquid nitrogen. CD4^+^ T cells were stimulated in vitro for 40 h with T cell‐activating αCD3/αCD28 Dynabeads (Thermo Fisher Scientific, 11132D) at 2 μL Dynabeads per 100 k cells. In some cultures, cells obtained from lean Y and O adults were treated with 0.3 μM Mitocur‐1 (Mtcur), 4 μg plasmid DNA pCDNA3‐STAT3‐S727D (Addgene:73364) or the backbone vector pCDNA3 (Addgene:20011) for 40 h. The cells were transfected utilizing Neon transfection system (ThermoFisher Scientific), according to manufacturer's instructions. All treatments were added to our standard culture media of RPMI with 5 mM glucose (normoglycemic), 10% heat‐inactivated FBS, and antibiotics. Supernatants were collected and stored at −80°C and shipped on dry ice to the University of Kentucky for performing Luminex bioplex assay to assess the levels of cytokines. Cells were assayed as outlined below.

### 2.2 Immunofluorescence

CD4^+^ T cells from Y and O adults and O adults treated in vitro with stimuli (αCD3/αCD28) plus 0.3 μM Mtcur were incubated or 40 h at 37°C in RPMI with 5 mM glucose. For intracellular cytokine (IL17F) staining, cell activation cocktail containing brefeldin A, phorbol 12‐myristate 13‐acetate and ionomycin (Biolegend) was added to cell for the last 6 h. The cells were collected and plated on coverslips in 12‐well plates coated with poly‐D‐lysine. The cells were briefly centrifuged (1200 rpm, 10 min), washed two times with 1X PBS and incubated in 4% paraformaldehyde for 30 min at RT. The coverslips were washed 2X with PBS and 0.1% triton X‐100 (PBST), and were blocked for at least 30 min in 5% BSA/PBST. Antibodies to CD45RA, CD45RO, CD27 (Biolegend), IL17F (Invitrogen, Bedford MA), TOM20, FIS1 (Santacruz Biotechnology), p‐STAT3 Ser 727, p‐STAT3 Tyr 705, STAT3, Mitofusin1 (Cell Signaling Technology), or FLAG tag (Millipore Sigma) were added at 1:50 dilution with incubation overnight at 4°C. Antibodies against cell surface proteins were added prior to permeabilization and incubated at RT for 1 hr. CD45RO cells were isolated utilizing MojoSort™ Human CD4 Memory T Cell Isolation Kit (Biolegend), CD45RA cells were isolated utilizing MojoSort™ Human CD4 Naïve T Cell Isolation Kit (Biolegend), according to manufacturers' instructions. The coverslips were washed 2X with PBST and incubated with fluorophore‐tagged secondary antibodies 1:500 (anti‐mouse Alexa 488 or anti‐ rabbit Alexa 647) (Rockland Immunochemicals, Thermo Fisher Scientific) for 2 h at RT. The coverslips were washed 2X with PBST and mounted on glass slides using Fluromount G (Southern Biotech). Cell imaging was performed using 63X oil immersion lens in a Zeiss LSM 800 confocal microscope. Approximately 5–7 fields/slide were imaged on *N* = 3–4 subjects per treatment, and data were analyzed using FIJI/Image J. Microscopy images were processed and analyzed as described (Kirber et al., 2007; Valente et al., 2017). Expression of proteins were reported as mean fluorescence intensity (MFI) and colocalization between two proteins were reported as Pearson's Colocalization Coefficient (PCC). PCC is a well‐established method that quantifies the degree of overlap between fluorescence in the channels. PCC is unaffected by changes to the offset and independent of gain. PCC has a range of +1 (perfect correlation) to −1 (perfect but negative correlation). PCC is not sensitive to differences in signal intensity between the components of an image caused by different labeling with fluorochromes, photobleaching or different settings of amplifiers (Adler & Parmryd, 2010; Conway et al., 2022)

### 2.3 Immunoblotting

Immunoblotting quantified protein expression as we published (Bharath et al., 2020; Conway et al., 2022). Briefly, 30 μL of 1X cell lysis buffer (Cell signaling technology) was added to 1 x 10^6^ cells and incubated on ice for 20 min. Cells were centrifuged at 13,000 rpm for 20 min and supernatant was collected. A Bicinchoninic acid assay (Thermo Fisher Scientific) assessed protein concentration. Fifteen μg protein was loaded onto polyacrylamide gels, and electrophoresis was performed at 100 V for 1 h. Transfer of protein to polyvinylidene difluoride (PVDF) membrane was performed at 35 V for 5 h. The membrane was blocked for 30 min at room temperature (RT) in blocking buffer containing 2% bovine serum albumin in TBST followed by overnight incubation at 4°C in the respective primary antibodies. The membrane was washed 2X with 1X TBST and incubated with the respective secondary antibodies for 2 h at RT, then imaged. Table 2 lists the antibodies used in this study. All primary antibodies were used at a dilution of 1:500 except β‐actin which was used at 1:10,000. All secondary antibodies were used at a concentration of 1:5000 except β‐actin which was used at 1:10,000. We quantified protein expression on western blots using Image studio lite software (Licor).

**TABLE 2:** Resource identification reagent or resource.

Antibodies	Source	Identifier
Mitofusin	Cell signaling technology	Cat# 14739, RRID:AB_2744531
OPA1	Cell signaling technology	Cat# 67589, RRID:AB_2799728
FIS1	Santa cruz Biotechnology	Cat# sc‐376,447, RRID:AB_11149382
Complexes antibody	Abcam	Cat# ab110411, RRID:AB_2756818
VDAC1	Santa cruz biotechnology	Cat# sc‐390,996, RRID:AB_2750920
STAT3 used at 1:500	Cell signaling technology	Cat# 4904; RRID, AB_331269
p‐STAT 705	Cell signaling technology	Cat# 9145, RRID:AB_2491009
p‐ STAT3 705	Cell signaling technology	Cat# 4113, RRID:AB_2198588
p‐STAT3 727	Cell signaling technology	RRID:AB_2737598
LDHA	Cell signaling technology	Cat# 3582, RRID:AB_2066887
FLAG	Sigma‐Aldrich	Cat#F1804; RRID, AB_262044
DRP1	Cell signaling technology	Cat# 14647, RRID:AB_2798554
efluor IL‐17 F	Thermo fisher scientific	Cat # 50–716‐942 RRID: AB_2574280
PE‐CD27	Biolegend	Cat # 393211 RRID: AB_2810586
Alexa −488 CD45RO	Biolegend	Cat # 304212 RRID: AB_528823
CD45RA	Biolegend	Cat # 304107 RRID: AB_314411
Anti‐TOM20 used at 1:50 for confocal microscopy	Santa cruz biotechnology	Cat# sc‐17,764, RRID:AB_628381
Anti‐mouse Alexa 488 used at 1:500	Rockland antibodies	Cat# 610–741‐124, RRID:AB_1057558
Anti‐rabbit Alexa 647 used at 1:500	Thermo fisher scientific	Cat# A‐21244, RRID:AB_2535812
Anti‐mouse IgG, HRP‐linked used at 1:5000	Cell signaling technology	Cat# 7076, RRID,AB_330924
Anti‐rabbit IgG, HRP‐linked used at 1:5000	Cell signaling technology	Cat# 7074, RRID:AB_2099233
Biological samples		
Lean normal glucose tolerant Y adults, CD4 ^+^ T cells Table 1	This paper	NA
Lean normal glucose tolerant O adults, CD4 ^+^ T cells Table 1	This paper	NA
Chemicals, peptides, and recombinant proteins		
Bovine serum albumin	Sigma aldrich	Cat# A8806
Mtcur‐1	Enamine	Cat# EN300‐188280
Antimycin A	Enzo life sciences	ALX‐380‐075‐M010
Oligomycin	Calbiochem	Cat # 495455
FCCP	Enzo life sciences	Cat # ML‐CM120‐0050
Rotenone	Enzo life sciences	Cat # ALX‐350‐360‐G001
Critical commercial assays		
Milliplex human Th17 25‐ plex kit	Millipore sigma	Cat# HT17MG‐14 K‐PX25
Complex I activity assay kit	Abcam	Cat# ab 109,721
Complex II activity assay kit	Abcam	Cat# ab 109,908
Legend Max IL17 A/F ELISA	Biolegend	Cat # 435807
Legend Max TNF α ELISA	Biolegend	Cat # 430207
Software and algorithms		
GraphPad prism version 7 for windows	GraphPad software	www.graphpad.com
Other		
Dynabeads human T‐Activator CD3/CD28 for T Cell activation	GIBCO life Technologies	Cat# 11132D
Human CD4 + Isolation kit	Miltenyi	Cat# 130–096‐533
CD4 + Isolation columns	Miltenyi	Cat# 130–042‐401
MojoSort™ Human CD4 Memory T cell isolation kit	Biolegend	Cat# 480063
MojoSort™ Human CD4 Naive T Cell Isolation Kit	Biolegend	Cat# 480041
Cell activation cocktail	Biolegend	423,303
RPMI, no glucose	Thermo fisher technologies	Cat# 11879020

### 2.4 Plasmid transfection

Plasmid extractions was performed using the ZymoPure II plasmid midiprep (Zymo Research Corporation) according to manufacturer's instructions. Four micrograms of the plasmid pCDNA3‐STAT3‐S727D or the empty vector was electroporated into CD4^+^ T cells using the Neon transfection system (Thermofisher Scientific). The transfection was confirmed by immunofluorescence as described above using antibodies to FLAG and phospho‐STAT3 Ser 727.

### 2.5 Extracellular flux analysis (Mito stress test)

The assay was performed according to the manufacturer's instructions and as we published (Bharath et al., 2020). CD4^+^ T cells were activated for 40 h along with the following treatments (± Mtcur, ± pCDNA3‐STAT3‐S727D). Then the cells (300,000/well) were plated onto wells of poly‐D‐lysine coated Seahorse XF HS mini plate (Agilent) in extracellular flux assay media (non‐buffered DMEM containing 5 mM glucose, 2 mM L‐glutamine, and 1 mM sodium pyruvate. Oxygen consumption rate (OCR) and extracellular acidification rate (ECAR) were measured using the mitochondrial stress test procedure for basal OCR followed by sequential addition of 3.5 μM oligomycin (Calbiochem), 1 μM fluoro‐carbonyl cyanide phenylhydrazone (FCCP) (Enzo Life Sciences), and 14 μM antimycin A (Enzo Life Sciences) with the XF HS mini Extracellular Flux Analyzer (Agilent) as previously described (Bharath et al., 2020; Nicholas et al., 2019).

#### 2.5.1 Mitochondrial network analysis (MiNA)

MiNA was performed and images were processed as described (Valente et al., 2017).

#### 2.5.2 Cell viability

Cell viability was assessed via trypan blue exclusion according to manufacturer's directions after the different treatments.

#### 2.5.3 Cytokine assay

Cytokine production was assessed in supernatants by Luminex multiplex assays (Milliplex human Th17 25‐plex kit, Millipore) as we published (Bharath et al., 2020; Conway et al., 2022; Ip et al., 2016). Outcomes from wells with <35 beads read for each analyte were excluded from analysis. Samples with >10% CV were removed from analysis. Plates were washed between incubations using a BioTek 406 Touch plate washer (BioTek) and read using the Luminex FlexMap 3D system (Luminex).

#### 2.5.4 Enzyme linked immunosorbent assay (ELISA)

Cytokine production by CD4^+^ naïve and memory cells was assessed by ELISA. CD4^+^ CD45RO^+^ cells were isolated utilizing MojoSort™ Human CD4 Memory T Cell Isolation Kit (Biolegend). CD4^+^ CD45RA^+^ cells were isolated utilizing MojoSort™ Human CD4 Naive T Cell Isolation Kit (Biolegend). Legend Max IL17A/F and TNFα kits (Biolegend) were utilized and the assay was performed according to manufacturers' instructions.

### 2.6 scRNA‐seq analysis

For the scRNA‐seq libraries, CellRanger (v.6.0.1) was used to perform sample‐demultiplexing, barcode processing and single‐nuclei gene‐UMI counting, which resulted in an expression matrix for each experiment by aligning to the reference GRCH38 using CellRanger with default parameters (Zheng et al., 2017). For initial quality‐control filtering, aligned cell and transcript counts from each experiment were processed by Seurat (Version 4.2) by removing cells containing a low abundance of detected genes (nFeature_RNA < =300), a relatively high abundance of UMIs (nCount_RNA > =10 k) and mitochondrial reads (> = 15% of total transcripts) (Hao et al., 2021). Expression data of cells passing these thresholds were then merged without any batch correction using merge in Seurat. To further check the quality of remaining cells, we estimated the doublets based on the merged data by applying the hybrid scores for doublet estimation generated from R package scds (Bais & Kostka, 2020). After the removal of likely doublets, we obtained a total of 39,227 cells. Expression data of the remaining qualified cells from every experiment were log normalized with the NormalizeData function and the top 2 K variable genes were identified with the FindVariableFeatures function. Data from all experiments were integrated again using Seurat's canonical correlation analysis (CCA) and Find Integration Anchors functions, to identify integration features and align the shared cell populations across experiments, which can correct for potential batch effects. The integrated data were then scaled with ScaleData function. Principal component analysis (PCA) was carried out with RunPCA function and the top 30 principal components (PCs) were retained. Clusters were identified with the FindClusters function by use of the shared nearest neighbor modularity optimization with a clustering resolution set to 0.5. Clusters with only one cell were removed. This method resulted in 16 clusters. To determine the cluster identity of Th17 effector cells, we checked the expression of five important cell markers, including CD4, CCR6, CCR4, NK1.1, and IL17. By visualizing the expression of all five markers across clusters in both TSNE plots and dot plots, two clusters (clusters 5 and 8) were annotated as Th17 effector cells (Figure S1A.B). Finally, a total of 5142 Th17 effector cells were identified, of which 3305 were from O adults and 1837 were from Y adults.

To evaluate the difference between samples from O and Y Individuals, differential gene expression analysis was conducted using Seurat FindMarker function with default Wilcoxon Rank Sum test. The parameter min.pct was set to 0.05 in order to include more genes that might not be widely detected. Genes with FDR <0.05 were considered as significant.

To visualize the expression of genes in mitochondrial complexes between O and Y, the expression of each interested genes was averaged across cells in each sample using Seurat Average Expression function. The expression patterns were visualized in heatmaps with z‐scores.

To calculate the differences in the expression of genes in Th17 clusters versus other CD4^+^ T cell clusters for aged and young groups, the differential expression test was first performed in Th17 cells compared to other cells, and the significant genes for mitochondrial structure and function in Y and O groups were identified with FDR <0.05. Then the fold changes at log2 scale of those significant genes were compared between Y and O groups using Wilcoxon signed‐rank test. To evaluate the difference in gene expression between the two Th17 clusters (clusters 5 and 8), the differential test was performed between Y and O in these two clusters independently. Genes expressed in at least 1% of the Y or O group were tested, and the overlap of genes that significantly differ in expression in the two clusters were identified.

### 2.7 Partial least squares modeling

To examine the differences between mitoSTAT3 inhibition or activation in T cells of O or Y individuals, respectively, we constructed predictive cytokine signatures using the linear, supervised multivariate mathematical modeling tool partial least squares discriminant analysis (PLS‐DA) (Wold et al., 1984) PLS maximizes multivariate covariation between predictors and sample identities when constructing latent variables (LVs), linear combinations of the predictors, allowing us to identify patterns of cytokine expression that predict outcome in this case, membership in age and/or treatment groups. PLS‐DA models were built in R using the ropls package (Thévenot et al., 2015) and models were orthogonalized to improve interpretability in models predicting the identity of two groups, maximally projecting covariation of the predictors with the sample identity to the first latent variable (Trygg & Wold, 2002). Four data subsets were processed for PLS‐DA by removing samples with missing cytokine readings and whole cytokines with a majority of readings below the detection limit (assigned 0 pg/mL) that did not correspond to sample identity. After generating PLS‐DA models with the data subsets, further variable selection was performed by extracting cytokines with a Variable Importance in Projection (VIP) score greater than 1 for the final model. A VIP score describes a variable's normalized contribution to the predictive accuracy of the model across all LVs (Galindo‐Prieto et al., 2014).VIP scores greater than 1 indicate a greater than average contribution to the model and were used to identify important differential cytokines. The data were mean‐centered and unit‐variance scaled prior to modeling. The number of LVs used in each model was chosen by repeated random subsampling cross‐validation, repeated 50 times for less than 15 samples or 100 times for greater than 15 samples, with 1/5 of the dataset left out to estimate prediction accuracy. Confidence was determined by a permutation test, where sample identities within the data set were randomly reassigned to the cytokine profiles. Models generated in the permutation test had the same number of LVs as the real model. Cross‐validation was performed as described to determine random accuracy and was repeated 100 times with new randomized sample identities for each iteration. Confidence was calculated by comparison with the mean and standard deviation of the distribution of random models' accuracy. PLS‐DA models containing one latent variable are visualized with two LVs in the score's plots, but only one latent variable was used in the calculation of accuracy, confidence, and VIP scores.

#### 2.7.1 Statistical analysis

Data are presented as mean ± standard error of the mean (SEM). Mann Whitney U test, Wilcoxon Rank Sum test, Kruskal‐Wallis with Dunn or one‐way ANOVA with Bonferroni post hoc tests were used depending on the data sets to compare means of the values. D'Agostino omnibus normality test was utilized to test the distribution. Significance was accepted when *p* < 0.05. Q‐test was used to detect outliers. Graph‐Pad Prism 10.0.2 (GraphPad Software) was utilized for statistical analysis and graphing.

## 3 RESULTS

### 3.1 Aging‐induced increase in mitochondrial localization of STAT3 was prevented by mitochondria targeted curcuminoid

**FIGURE 1:**
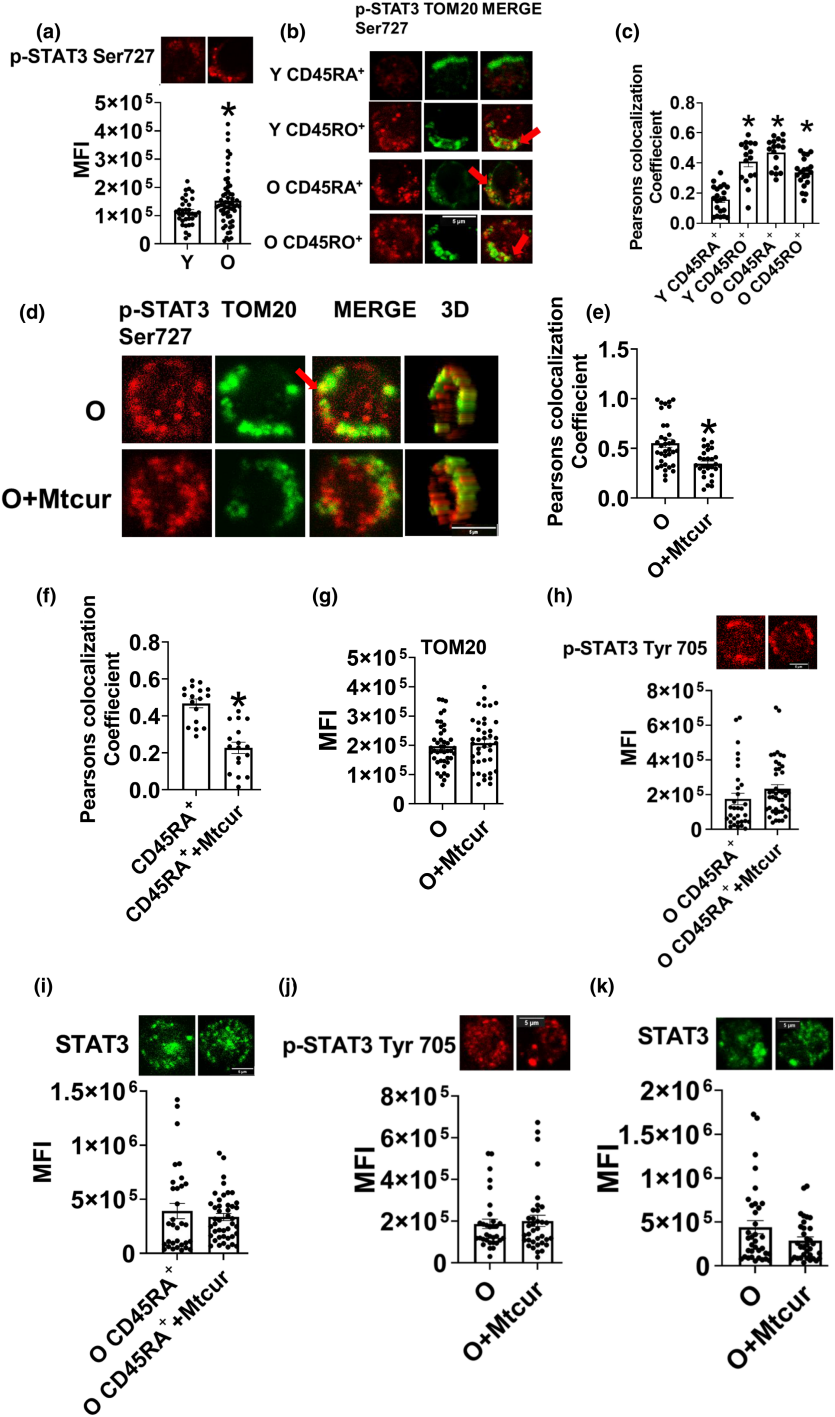
Mitochondrial localization of STAT3 increases with age. Cellular localization of STAT3 in T cells from younger (Y) and older (O) adults. Expression (a) and mitochondrial localization of p‐STAT3 Ser727, representative images (b) and quantification (c). Mitochondrial localization of p‐STAT3 Ser727 in O cells ±0.3 μM Mtcur treatment, representative images (d) and quantification (e). localization of mitoSTAT3 in naïve cells from O adults (f), mitochondrial outer membrane protein TOM20 (g), tyrosine 705 phosphorylated STAT3 (h) and STAT3 protein in naïve cells from O adults (i) tyrosine 705 phosphorylated STAT3 (j) and STAT3 protein (k) in CD4^+^ T cells from O adults± Mtcur treatment. *N* = 3–4, a–i. *N* = 3–4 indicates cells were obtained from either three or four individuals for each condition. Microscopy data are represented as fields of view. At least and 5–7 fields per slide were imaged at 63Xmagnification with oil immersion, on a Zeiss LSM 800 confocal microscope. In fields where, numerous cells were observed the mean fluorescence intensity of 3–4 cell groups were plotted as a single dot. Images were processed as described in methods and brightness was adjusted to improve clarity. Mann Whitney Test and Wilcoxon matched‐pair signed rank test, *p < 0.05 versus Y or O.

Our earlier work showed that aging induces the production of Th17 cytokines which are generally considered proinflammatory and implicated in the onset and progression of multiple age‐associated diseases including autoimmunity and malignancies (Bharath et al., 2020). Our work pinpointed the existence of multiple mechanisms that promote Th17 inflammation and the presence of STAT3 in the mitochondria emerged as a viable mechanism that promotes inflammation with age. In this study, we sought to evaluate the impact on mitoSTAT3 on T cell mitochondrial structure, dynamics, function and cytokine production. STAT3 phosphorylation, required for its mitochondrial translocation, was assessed in CD4^+^ T cells from Y and O adults. Significantly more Ser 727 phosphorylated STAT3 was observed in CD4^+^ T cells from O adults (Figure 1a). Additionally, we observed more p‐STAT3 Ser727 localization in the mitochondria of both the naïve (CD45RA^+^) and memory (CD45RO^+^) population of T cells of O adults, whereas only memory cell population of Y adults had higher mitoSTAT3, as shown by colocalization between p‐STAT3 Ser727 and mitochondrial translocase of outer membrane; TOM20 (Figure 1b representative images & c quantification). MitoSTAT3 was also assessed in memory cells that are CD45RO^+^ CD45RA^−^ CD27^+^ and CD45RO^+^ CD45RA^−^ CD27^−^ and no difference was observed^−^ (Figure S4a, bars 1 vs. 2). CD45RO^+^ CD27^−^ cells are effector cells that display high antigen recall response whereas CD45RO^+^ CD27^+^ cells are intermediate memory population that lacks high antigen recall response. To understand the physiological relevance of mitoSTAT3 on T cell function, we utilized Mtcur‐1(Mtcur), a mitochondria‐targeted curcuminoid which is known to limit mitoSTAT3 in other cell types (Erlich et al., 2018) (Erlich et al., 2014; Pavlyuchenkova et al., 2023) and in immune cells (Conway et al., 2022). Stimulation of T cells from O adults in the presence of Mtcur for 40 h significantly reduced mitochondrial localization of p‐STAT3 Ser 727 (Figure 1d representative images & e quantification), compared to stimulation in the absence of Mtcur. The reduction was specifically in the naïve T cells of O adults (Figure 1f) but not in the memory cell (Figure S4a, bars 3 & 4). The expression of p‐STAT3 Ser 727 after Mtcur treatment was also lower only in naïve cells from O adults but not in memory (Figure S4b, bars 1 vs. 3 & 2 vs. 4). Mtcur did not change the expression of mitochondrial protein TOM20 (Figure 1g) or phosphorylation of STAT3 at tyrosine 705(p‐STAT3 Tyr705) in naïve CD4^+^ T cells (Figure 1h) or total CD4^+^ T cells(Figure 1j), or STAT3 protein expression in naïve CD4^+^ T cells (Figure 1i), or total CD4^+^ T cells (Figure 1k). Cell viability was not affected by treatment with Mtcur assessed by trypan blue assay (data not shown). Collectively, these data show that aging induces mitochondrial translocation of STAT3, more specifically in the naïve cells and Mtcur limits mitoSTAT3 without affecting either total STAT3 or phosphorylation of the protein at the tyrosine residue.

### 3.2 Reducing mitochondrial localization of STAT3 prevents aging‐induced changes in mitochondrial structure

**FIGURE 2:**
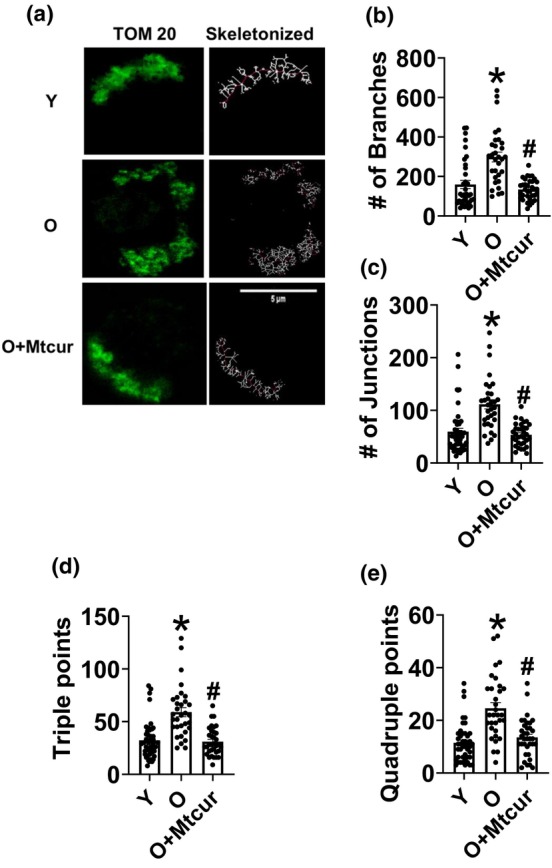
Limiting mitoSTAT3 reduces mitochondrial branching. Mitochondrial network analysis was performed on T cells from Y and O adults ± in vitro Mtcur treatment. Representative images (a) the number of branches (b) number of junctions (c). Triple points (d) and quadruple points (e). *N* = 3–4, a–e. At least 5–7 fields per slide were imaged at 63X magnification with oil immersion, on a Zeiss LSM 800 confocal microscope. Images were processed as described in methods and brightness was adjusted to improve clarity. Kruskal‐Wallis test, *p < 0.05 versus Y, #*p* < 0.05 versus O.

We performed a simple MiNA using FIJI to evaluate if reducing mitoSTAT3 has an impact on mitochondrial structure. MiNA was performed as described (Valente et al., 2017). Preventing mitoSTAT3 reduced aging‐induced branching and reticulation of mitochondria and restored the arrangement similar to T cells from Y adults. Representative images are shown in (Figure 2a), quantification of the number of branches, (filaments attached to branch points), junctions, (originating points for branches), triple points, (number of points where three mitochondrial branches are attached) and quadruple points, (number of points where at least four mitochondrial branches are attached) (Figure 2b–e). Mtcur lowered all variables in cells from O adults to the levels observed in Y adults. We conclude that T cells from Y adults have a compact mitochondrion with less branching, and age‐ associated increases in mitochondrial branching and reticulation is abrogated by limiting mitoSTAT3.

**FIGURE 3:**
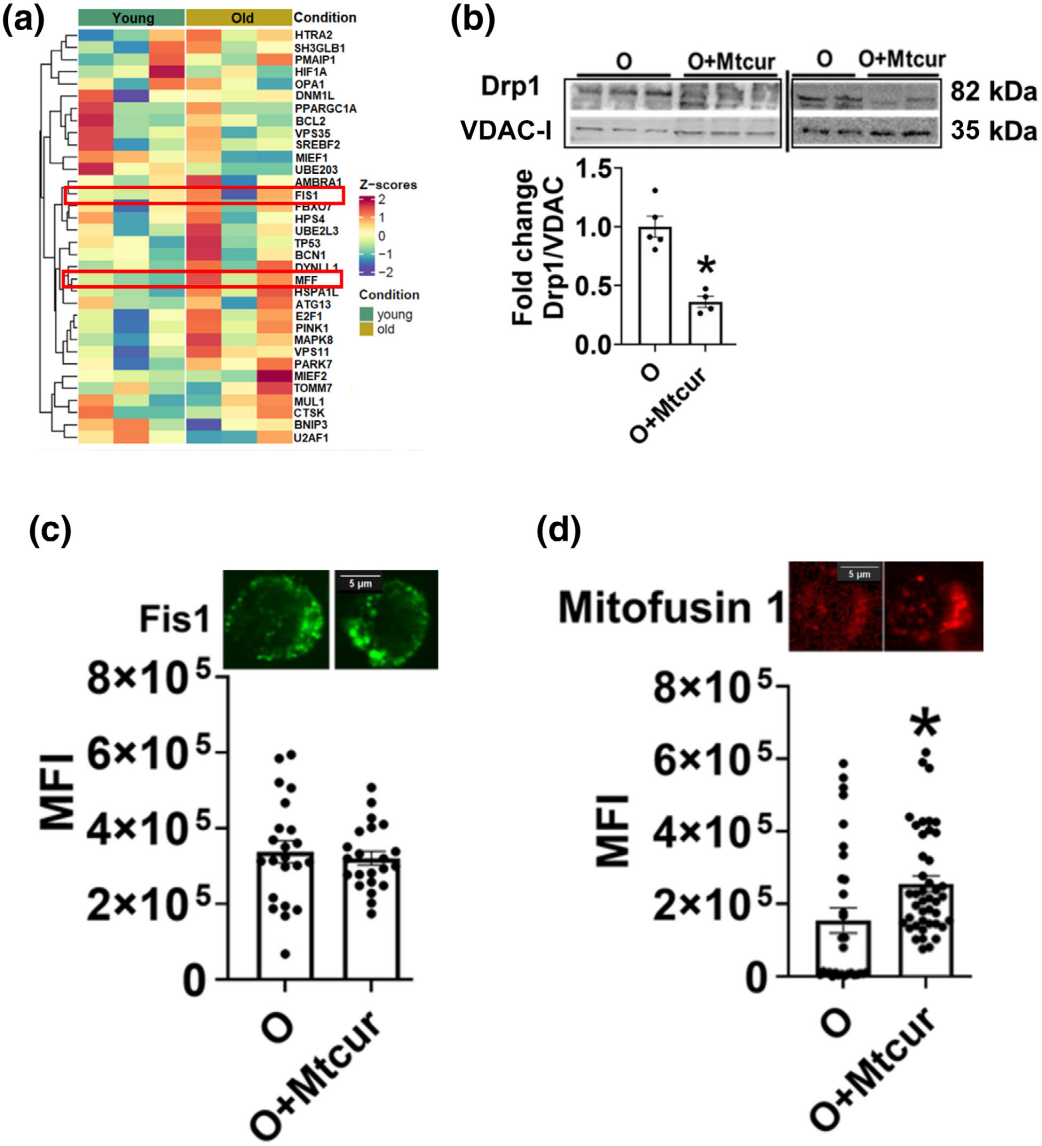
Limiting mitoSTAT3 prevents fission and promotes mitochondrial outer membrane fusion. Heatmap of genes involved in mitochondrial organization (a) Expression of Drp1 (b), Fis1(c) and mitofusin 1(d) in T cells from O adults. *N* = 3, a, c, d. *N* = 4–5, b. For microscopy experiments, at least 5–7 fields per slide were imaged at 63X magnification with oil immersion, on a Zeiss LSM 800 confocal microscope and the mean fluorescence intensity are plotted. Brightness of images were adjusted to improve clarity. Wilcoxon matched‐pair signed rank test, *p < 0.05 versus O.

We evaluated the mitochondrial fusion and fission markers to gain a better understanding of the role of mitoSTAT3 in modulating the mitochondrial dynamics in T cells from O adults. Single cell RNA sequencing showed that mitochondrial fission protein Fis 1 and mitochondrial fission factor MFF expression were numerically higher in T cells from O adults compared to Y adults (Figure 3a). Figure S1a, shows the TSNE plots of the clusters identified as Th17 cells and the density plots showing Th17 cell specific gene expression markers used to identify the clusters (Figure S1b). The percentage of Th17 cells to other CD4^+^ T cells (Figure S1c). Mitochondrial genes showing significant difference (FDR <0.05) between Y and O adults in Th17 cells vs other CD4^+^ T cells are shown in Figure S1d. Since Fis 1 and MFF recruit the mitochondrial fission protein Drp1 to the mitochondria, we assessed expression of Drp1 in T cells from O adults. Mtcur treatment of cells from O adults reduced the expression of Drp‐1 (Figure 3b). Surprisingly, fis1 protein expression was not altered by Mtcur treatment (Figure 3c), but Mtcur increased expression of outer mitochondrial membrane fusion protein mitofusin‐1(Figure 3d). The inner mitochondrial membrane fusion protein Opa1 was unchanged by Mtcur and supported Sc‐RNA sequencing data, which showed no difference in Opa1 between Y and O adults (Figure 3a). Although confocal microscopy does not rigorously delineate mitochondrial ultra‐structure, z‐stacked images and quantitative analysis nonetheless revealed a branched and reticular mitochondrial morphology, rather than punctuate and fissioned mitochondria, in T cells from O adults. Overall, limiting mitoSTAT3 decreased the number of mitochondrial branches and branching points, concomitant with increasing the outer membrane fusion protein Mfn1, to reduce reticulation, and restore mitochondrial morphology to that of T cells from Y adults.

### 3.3 Aging promotes expression and activity of mitochondrial complex II in CD4 ^+^ T cells

**FIGURE 4:**
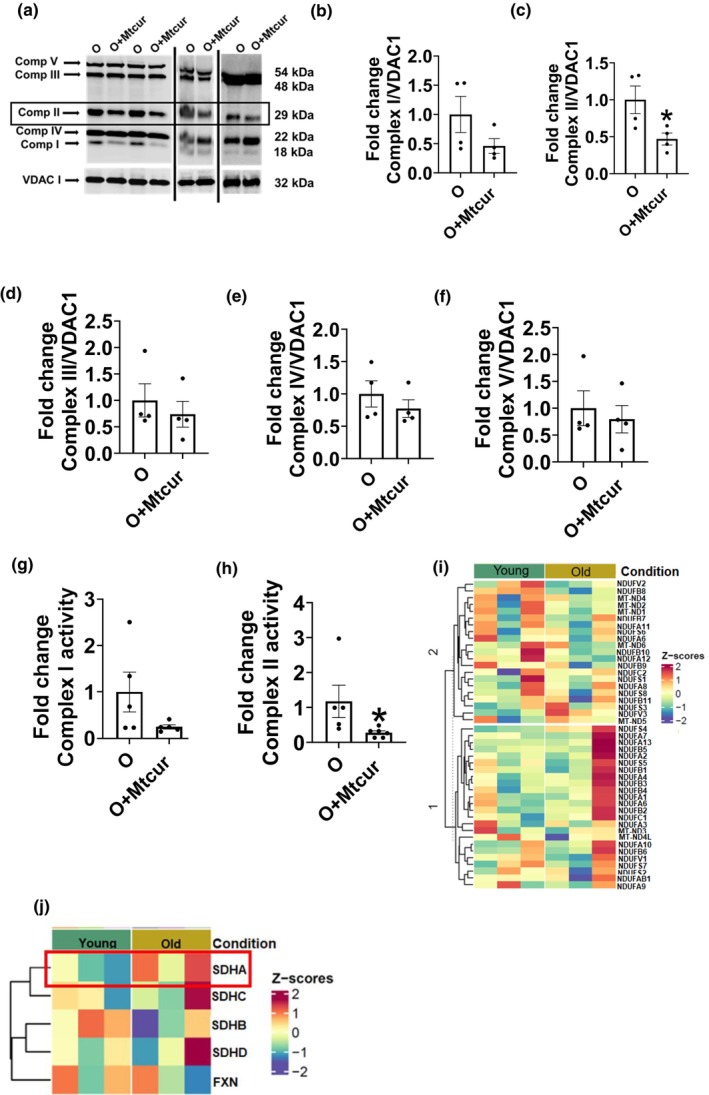
Mtcur reduces T cell mitochondrial complex II expression and activity. Expression of mitochondrial respiratory chain complex proteins, representative blot (a), quantification of complex I (b), complex II (c), complex III (d), complex IV (e) and complex V (f). Mitochondrial respiratory complex activities, complex I (g), complex II (h). Heatmap showing gene expression of mitochondrial complex I (i), and complex II (j) subunits in T cells from Y and O adults. *N* = 4, a–f. *N* = 5–6, g, h. *N* = 3, i, j. Wilcoxon matched‐pair signed rank test, *p < 0.05 versus O.

Since a prominent function of mitochondria is the generation of ATP, we evaluated if energy generation by mitochondria, in addition to structure, are altered by mitoSTAT3. The expression of mitochondrial complex proteins measured via immunoblotting showed that Mtcur treatment reduced the expression of complex II protein (Figure 4a; representative blots, Figure 4c, quantification). Mtcur did not change the expression of complexes I, III, IV & V (Figure 4a,b; d–f). Mtcur significantly lowered activity of complex II (Figure 4h) but did not alter the activity of complex I (Figure 4g) as measured by a colorimetric activity assay (Abcam, Waltham, MA). Sc‐RNA seq data shows differential changes in mitochondrial complex I genes in CD4^+^ T cells between Y and O in (Figure 4i) and complex II gene succinate dehydrogenase A (SDHA) was higher in CD4^+^ T cells from O compared to Y adults (Figure 4j). The differences in the genes of the mitochondria and other genes that were significantly different (FDR <0.05) in Th17 effector cells versus other CD4^+^ T cells from each donor of Y and O are shown in Figure S1d. Differential expression of genes between Y and O adults in the two Th17 effector clusters 5 & 8, are depicted in Figure S2a,b. These data collectively show that CD4^+^ T cells from O adults have higher mitochondrial complex II expression and activity, both of which are significantly blunted by Mtcur.

### 3.4 Pharmacological and genetic manipulation of p‐STAT3Ser727 alters mitochondrial oxygen consumption

**FIGURE 5:**
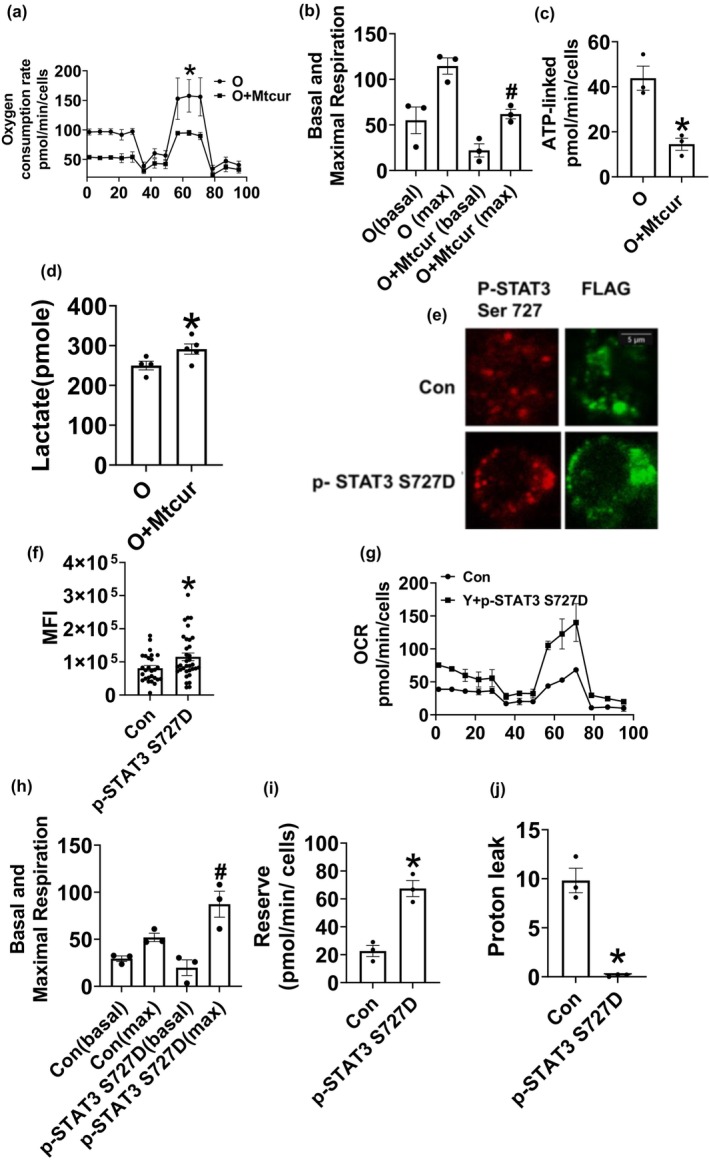
Limiting mitoSTAT3 reduces T cell OXPHOS. Mitochondrial oxygen consumption rate (a) and (b), ATP production (c) and lactate production (d). Expression of constitutively Ser727 phosphorylated STAT3 in T cells from Y adults, representative images (e), quantification (f). Mitochondrial oxygen consumption rate in Y cells expressing p‐STAT3S727D (g) and (h), reserve capacity (i) and proton leak (j). N = 3 a‐c, e‐j, N = 4–5 d. For microscopy experiments, at least 5–7 fields per slide were imaged at 63X magnification with oil immersion, on a Zeiss LSM 800 confocal microscope. Brightness was adjusted to improve clarity. Wilcoxon matched‐pair signed rank test, *p < 0.05 vs. O or Con, #*p* < 0.05 versus O (max) or Con (max).

**FIGURE 6:**
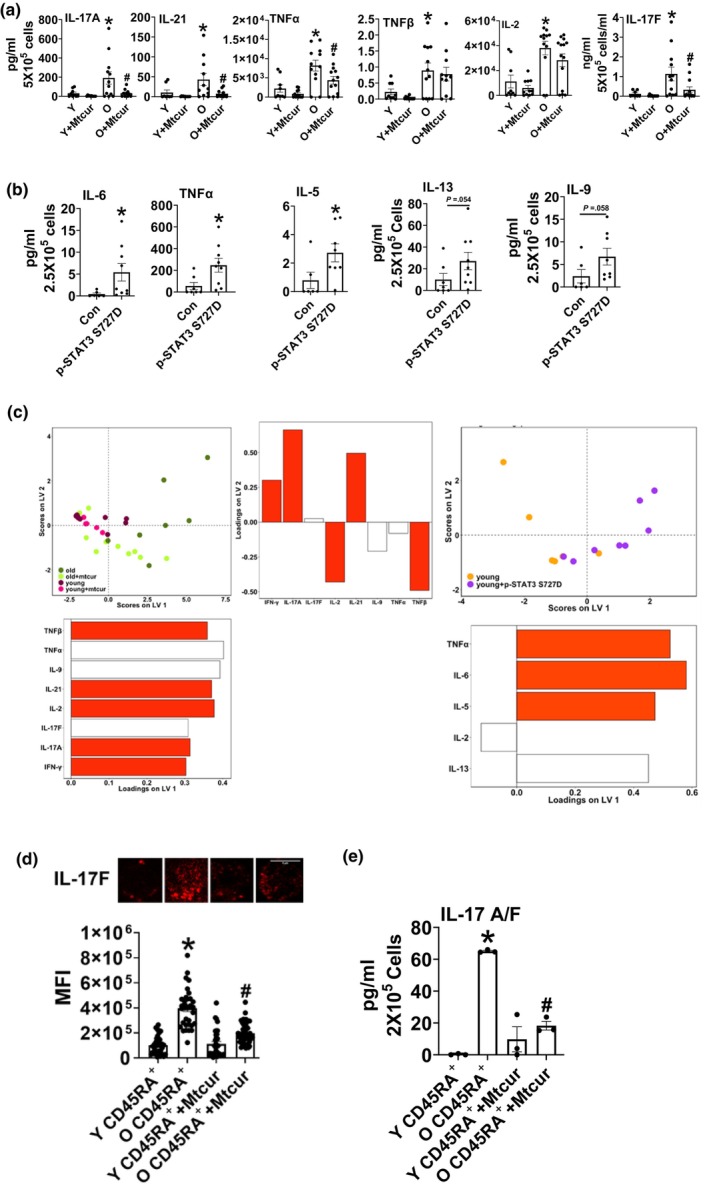
MitoSTAT3 impacts cytokine production. Cytokine production was assessed in T cells from Y and O adults ± in vitro Mtcur treatment (a) and in Y cells after transfection with either empty vector or p‐STAT3 S727D (b). PLS‐DA scores plot (top) and Y and O ± Mtcur, (3 LV, accuracy: 54.7%, confidence: 100%) and Y vs Y + p‐STAT3 S727D (1 LV, accuracy: 73%, confidence: 92.2%) (c). Red cytokine loadings signify cytokines with a VIP score >1 and thus most influential in distinguishing the conditions. Each point represents a single individual. Intracellular staining of IL‐17 F in naïve T cells (d) and IL‐17 A/F production in naïve cells (e). N = 8–12, a, N = 7–9, b, N = 7–8 (O ± Mtcur) and N = 6–8 (Y and Y + p‐STAT3 S727D), c. N = 3 d & e a. Either Kruskal‐Wallis or One‐way Anova with Bonferroni post hoc was performed for univariate analysis. D'Agostino omnibus was utilized to test the normality of the data. b. Wilcoxon matched‐pair signed rank test, *p < 0.05 versus O or Con.

Electron transport chain activity and oxidative phosphorylation (OXPHOS) are important for mitochondria to generate ATP. We confirmed (data not shown) that the OCR, an indicator of OXPHOS, of T cells from O adults is high, in line with our previous work (Bharath et al., 2020). Mtcur significantly reduced high age‐related OCR (Figure 5a,b) and ATP linked respiration (Figure 5c), while increasing lactate production (Figure 5d), without increasing the expression of lactate dehydrogenase (data not shown). To independently assess the involvement of mitoSTAT3 in regulating mitochondrial OXPHOS, CD4^+^ T cells from Y adults were transfected with a phosphomimetic p‐STAT3 S727D, which results in the constitutive phosphorylation at Ser 727 (Figure 5e representative images showing the expression p‐STAT3 S727D(red) and FLAG tag (green) & f quantification;). CD4^+^ T cells from Y adults expressing p‐STAT3 S727D had higher OCR (Figure 5g,h) and reserve capacity (Figure 6i), but significantly lower proton leak (Figure 6j) compared to control cells. Collectively, these data show that mitoSTAT3 favors OXPHOS.

### 3.5 Modulating mitoSTAT3 prevents aging‐induced CD4 ^+^ T cell proinflammatory cytokine production

We measured the effect of modulating mitoSTAT3 on T cell cytokine production by adding Mtcur along with CD3/CD28 treatment of T cells from Y and O adults, or T cells from Y adults expressing p‐STAT3 S727D. Mtcur reduced the production of Th17 cytokines IL‐17 A, IL‐17F, IL‐21, which we previously showed dominate aging‐related inflammation, in cells from O adults (Bharath et al., 2020). Mtcur also reduced production TNF α, (Figure 6a). Additional cytokine outcomes are shown in Figure S3a. The percentage of T effector cells in O adults ± Mtcur is shown in (Figure S3b). IL‐17 production assessed via intracellular staining for IL‐17F and ELISA assay for IL17‐A/F heterodimer in naïve CD45RA^+^ and memory CD45RO^+^ cell population from Y and O adults. Naïve cells from O adults produced higher amounts of IL‐17F (Figure 6d) and IL17A/F (Figure 6e) than naïve cells from Y adults and Mtcur reduced the production of the cytokine in naïve T cells from O adults. Memory CD45RO^+^ cells showed no difference between Y and O and after Mtcur treatment Figure S3c (IL17 A/F heterodimer), which supports the findings that robust increase in mitoSTAT3 occurs in the naïve cells in O adults and Mtcur does not impact memory cell cytokine production in the absence of naïve cells. Expressing p‐STAT3 S727D in T cells from Y adults increased Th17 supporting cytokines such as TNFα and cytokine IL‐6, but not cytokines IL‐17 A and IL‐17F (Figure 6b and Figure S5a). TNF‐α production was higher in T cells from O adults and in Y adults expressing p‐STAT3 S727D, while it was significantly blunted when mitoSTAT3 was limited. Exogenous p‐STAT3 S727D localization in the mitochondria remained refractory to Mtcur treatment (Figure S4c,d) thus indicating that exogenous and constitutive expression of p‐STAT3 Ser 727 is not identical to endogenous expression and this variability could also explain exogenous p‐STAT3 S727D promoting Th17 supportive cytokines and not Th17 cytokines. Investigating the speculated differences would require proteomic analyses that go beyond the current scope.

Partial least square discriminant analysis (PLS‐DA) models combined all cytokines from one sample into a multi‐dimensional value for inflammation (Figure 6c). Mtcur treatment did not affect the cytokine production in T cells from Y adults in a predictable way. The variable importance prediction scores (VIP) ranked Th17 cytokines as important for separating the data clouds between O and O after Mtcur treatment, whereas Th17 supporting cytokines TNFα and IL‐6 but not Th17 perse were identified as most important for differentiating Y and Y cells expressing p‐STAT3 S727D. Thus, the induction of TNFα pinpoints the mechanistic link between mitochondria and effector function of the T cells and also the existence of other mechanisms important for Th17 inflammation.

## 4 DISCUSSION

Our data show that mitochondrial localization of STAT3 increases in CD4^+^ T cells with age and changes structure and function of mitochondria to impact T cell cytokine production. In vitro treatment of cells with mitochondria‐targeted curcuminoid, Mtcur‐1, an inhibitor of mitoSTAT3 pointed to the involvement of mitoSTAT3 in mitochondrial complex II function and mitochondrial dynamics.

Age‐related changes in the mitochondrial network have precedent for changes in cell function. A branched intermyofibrillar mitochondria observed in aged mice was associated with sarcopenia (Leduc‐Gaudet et al., 2015) Furthermore, a three dimensional and quantitative electron microscopy revealed mitochondrial branching and formation of a complex nanotunnels in skeletal muscles of humans with mutations in mtDNA (Vincent et al., 2019). Although age‐relatedness of this outcome was not queried, the authors concluded that low level of stress or mutational heteroplasmy promote mitochondrial reticulation, and increased nanotunnel formation and that beyond a functional threshold the network undergoes fragmentation (Leduc‐Gaudet et al., 2015) (Shutt & McBride, 2013). Taking into consideration the observations from the literature, it is likely that the highly reticular mitochondrial structure is the result of chronic but low‐level stress that accompanies aging to promote the reticulation of mitochondria in T cells.

Alteration of proteins involved in mitochondrial structure can perturb mitochondrial function, especially the activity of the electron transport chain (ETC) (Liesa & Shirihai, 2013). Succinate dehydrogenase (SDH) or complex II, which was higher in O adults and Mtcur sensitive, is embedded in the mitochondrial inner membrane and catalyzes the activity of TCA cycle and the ETC. SDH dysfunction is linked to failure of cells to undergo apoptosis and generate reactive oxygen species (ROS), (Bezawork‐geleta et al., 2020), but these possibilities have not been tested in T cells. In LPS‐ stimulated macrophages, increased succinate dehydrogenase activity, succinate oxidation and ROS resulted in proinflammatory gene expression, while inhibition of succinate oxidation promoted anti‐inflammatory outcomes (Mills et al., 2018). While it is interesting to speculate the existence of a direct interaction between mitoSTAT3 and complex II in immune cells, this is unlikely, based on the stoichiometric relationship between the complexes and STAT3 observed in isolated cardiac mitochondria (Phillips et al., 2010). It is likely that the regulation between mitoSTAT3, SDH activity and oxidative phosphorylation is due to signaling events, that warrant further investigation.

In our previous studies (Bharath et al., 2020) and data herein we show that CD4^+^ T cells from O adults have heightened basal and maximal OCR indicating that the cells are utilizing mitochondrial oxidative phosphorylation. Whereas activated T cells from lean Y adults have higher glycolytic activity and are reported to have higher levels of pyruvate, lactic acid, and express high levels glycolysis pathway proteins (Shan et al., 2020). The OXPHOS dependence of T cells from O adults is functionally relevant because studies show T cell OXPHOS supports pathogenic Th17 cell differentiation. (Hong et al., 2022; Zambrano‐zaragoza et al., 2014).

Limiting mitoSTAT3 prevented the production of the cytokines TNFα, IL‐17A, IL‐17F and IL‐21 that are generally implicated in promoting inflammatory conditions. Combinatorial analysis utilizing the Partial Least Square‐ Discriminant analysis (PLS‐DA) and VIP score identified all the above‐mentioned cytokines as important in defining the O group and were abrogated by Mtcur treatment. Overexpression of constitutively phosphorylated STAT3 in T cells from Y adults increased the expression of TNFα and IL‐6. Although we did not see an induction of Th17 cytokines. It is important to note that both IL‐6 and TNFα are known to induce IL‐17 production in T cells. (Korn et al., 2008) (Pesce et al., 2022) A few reasons can be proposed for lack of IL‐17 production after constitutive exogenous expression of p‐STAT3 S727D. Homeostasis mechanisms are robustly operational in T cells from Y adults but are dysregulated in T cells from O adults, thus Th17 production may be induced more readily in cells from O adults when compared to cells from Y adults, (ii) The dose and duration of mitoSTAT3 and thus the strength of the signal might be important in production of Th17 cytokines, that can vary between the Y and O. While we saw the induction of Th‐17 supporting cytokines TNFα and IL‐6, it might take a longer for cells expressing exogenous STAT3 to produce some cytokines as the result of changes induced by the experimental procedure, (iii) Mtcur, a pharmacological agent, might have had effects on other pathways, which may affect cytokine production. Mtcur is known to have redox regulatory effects which was demonstrated in other cell types, however our data showed that Mtcur did not alter peroxide or mitochondrial superoxide production in T cells from O adults iv) we observed that unlike endogenous p‐STAT3 Ser 727, exogenous and constitutive expression of p‐STAT3 S727D was refractive to Mtcur thus showing that exogenous expression might be similar but is not identical to endogenous p‐STAT3 ser 727 (v) most importantly, there could be additional key players that are important for induction of pathogenic inflammation which are not yet know, and is a part of our ongoing investigation. Interestingly, when compared to T cells from Y adults, T cell from O adults had significantly higher mitoSTAT3 in naïve cells, whereas the memory component was comparable between Y and O. Mtcur effect was also prominent in the naïve population in T cells from O adults. Additionally, Mtcur did not alter the expression of STAT3 or the phosphorylation at residue Tyr 705, which is indispensable for nuclear transport and transcriptional function of STAT3. However, other studies have shown that once‐activated nuclear STAT3 can be inactivated and proceed to Tyr 705 dephosphorylation and nuclear export and this processes of STAT3 inactivation are known to be regulated by phospho‐STAT3 S727 (Wakahara et al., 2012; Yang et al., 2019). The detailed analysis of the mechanisms that promote mitoSTAT3 induction in aging naïve T cells, the association between mitoSTAT3 and SDH and the overall outcomes of immune response during aging are part of ongoing investigations.

Collectively our study provides evidence for the existence of mechanistic links among mitochondrial STAT3, T cell mitochondria, and Th17 proinflammatory cytokine production.

### 4.1 Limitations and future studies

While we documented the mitochondrial structural and functional changes and its association to mitoSTAT3, we are yet to assess the detailed signaling events and mechanisms which contributed to the functional outcomes. Mtcur, a pharmacological agent may have impacted other mechanisms and pathways thus influencing T cell function. The regulation of mitochondrial complexes by mitoSTAT3 is documented, however, studies show a direct interaction between STAT3 and mitochondrial complexes may or may not be possible due to the stoichiometric differences between the complexes and STAT3, thereby pointing to indirect effects, which must be further investigated.

## AUTHOR CONTRIBUTIONS

Conceptualization—Leena P. Bharath. Methodology—Leena P. Bharath, Investigation—Emelia Zukowski, Marco Sannella, Jack Donato Rockhold, Gabriella H. Kalantar, Nasun Hah, Ling Ouyang, Tzu‐Wen Wang, Sara SantaCruz‐Calvo, Lyanne Murphy, Heather Marszalkowski, Kaleigh Gibney, and Leena P. Bharath. Formal Analysis‐ Emelia Zukowski, Marco Sannella, Jingting Yu, Elizabeth A. Proctor, Madison K. Kuhn. Supervision—Leena P. Bharath. Writing‐ Leena P. Bharath, Editing‐Barbara S. Nikolajczyk, Hatice Hasturk, Micah J. Drummond, Funding‐ Leena P. Bharath, Barbara S. Nikolajczyk, Resources—Hatice Hasturk.

## FUNDING INFORMATION

This work was supported by R15AG068957 (LPB), pilot award from the San Diego Nathan Shock Center of Excellence in the Basic Biology of Aging NIH P30AG068635 (LPB) and R56AG06985 (BSN), T32NS115667 (MKK). R01AG076075 (MJD) and supported by the Pasini Fellowship (LPB), College of Health Sciences Faculty Development grant (FDG) (LPB), and Sakowich Center for Undergraduate Research and Creative Activities grant (SCURCA), Merrimack College (LPB), College of Health Sciences, Merrimack College and Barnstable Brown Diabetes Center, University of Kentucky (BSN).

## 5 CONFLICT OF INTEREST STATEMENT

All authors have no conflict of interest to declare

## Supporting information


**Appendix S1:** Supporting Information

## Data Availability

The data that support the findings of this study are available from the corresponding author upon reasonable request. Single cell RNA sequencing data is available on Gene Expression Omnibus (GEO), accession number GSE 241492.
