# Supplementary material for: STAT3 modulates CD4 + T mitochondrial dynamics and function in aging
Source: Aging Cell. 2023 Oct 13;22(11):e13996. doi: 10.1111/acel.13996 (PMC10652300; doi:10.1111/acel.13996)
Supplement: Supplementary file 1 — Appendix S1: Supporting Information [file ACEL-22-e13996-s001.pdf]

Supplementary Figure 1. Plots showing Th17 distribution

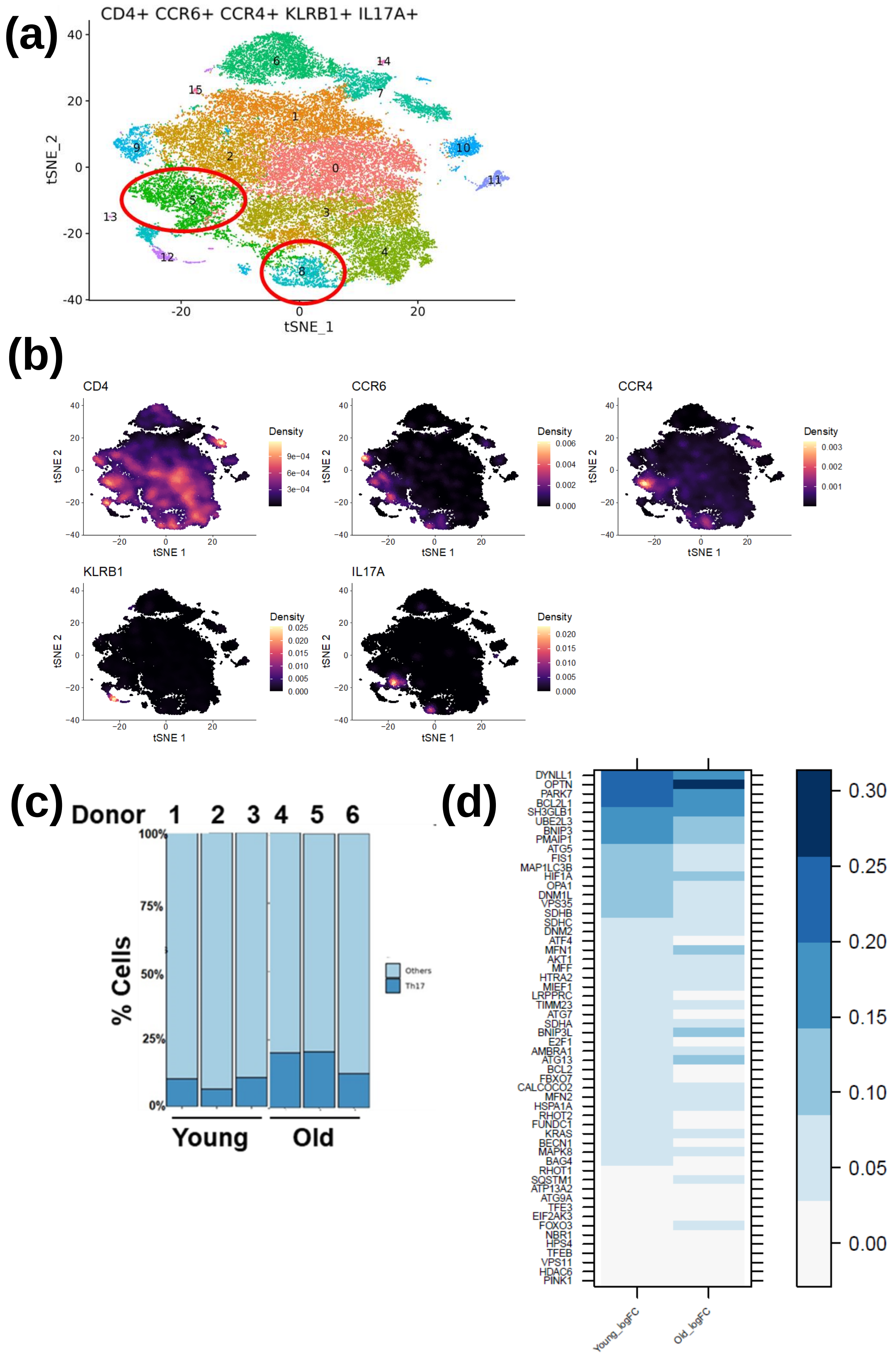

Supplementary Figure 1: Annotation of cells after Sc-RNA sequencing showing Th17 clusters **(a)** and density plots showing Th17 cell specific gene expression markers used to identify the clusters **(b)**, percentage of Th17 effector cells vs. other $CD4^+$ T cells in donors **(c)** Genes showing significant difference between Y and O adults in Th17 cells vs other $CD4^+$ T cells (p=0.001, Wilcoxon signed-rank test). Plot shows log2 fold change (FDR<0.05), **(d)**, N= 3 a-d, cells were obtained from three young adults and three older adults.

Supplementary Figure 2. Differential expression of genes in Th17 subset

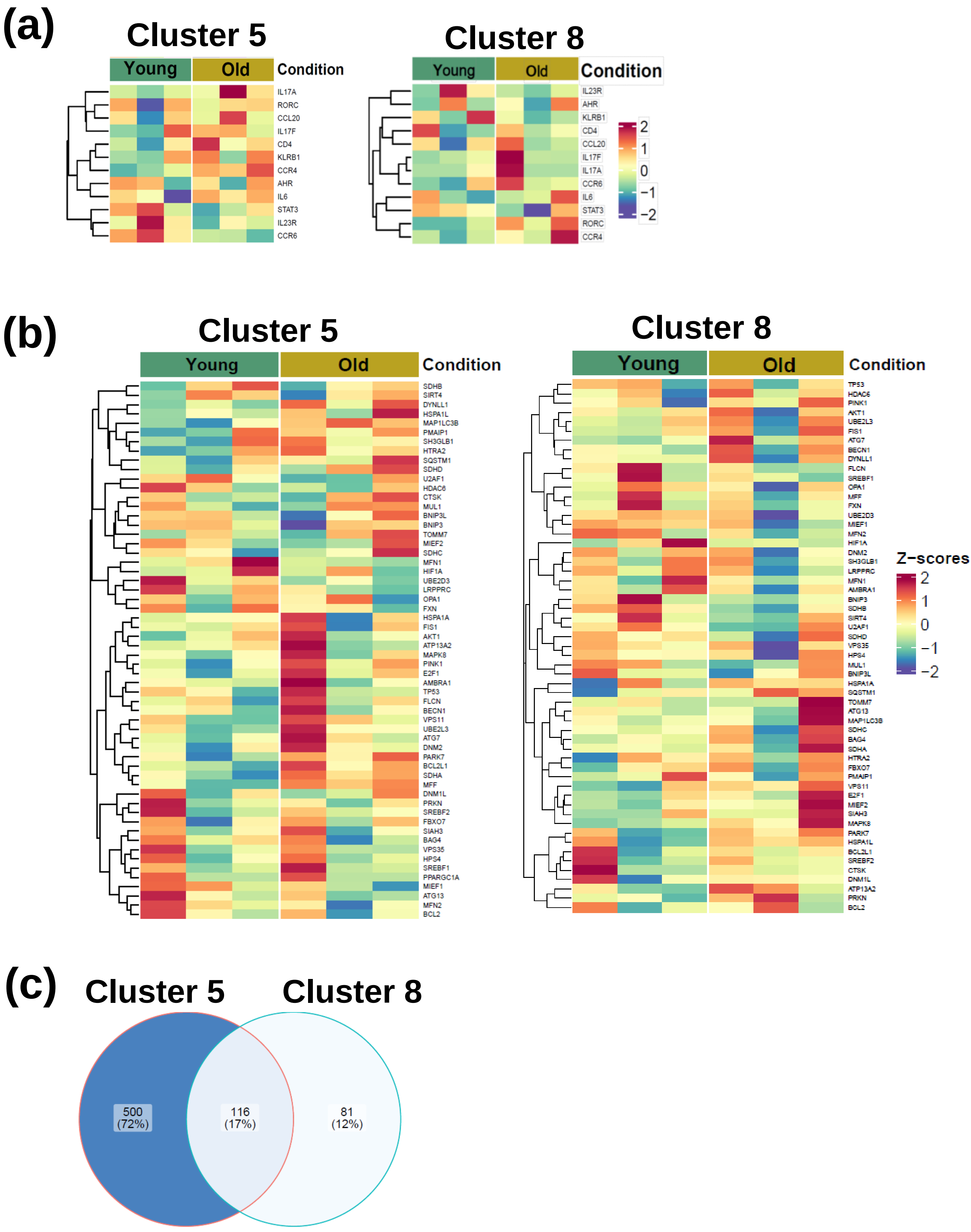

Supplementary Figure 2: Differential expression (DE) of genes between Y and O adults in cluster 5 vs cluster 8. Th17 effector genes **(a)**, mitochondrial genes **(b)**, plot showing the percentage of DE genes that overlap in the two clusters **(c)** N= 3 a-c, cells were obtained from three young adults and three older adults. p=0.001, Wilcoxon signed-rank test.

Supplementary Figure 3. Cytokine production after mitoSTAT3 inhibition.

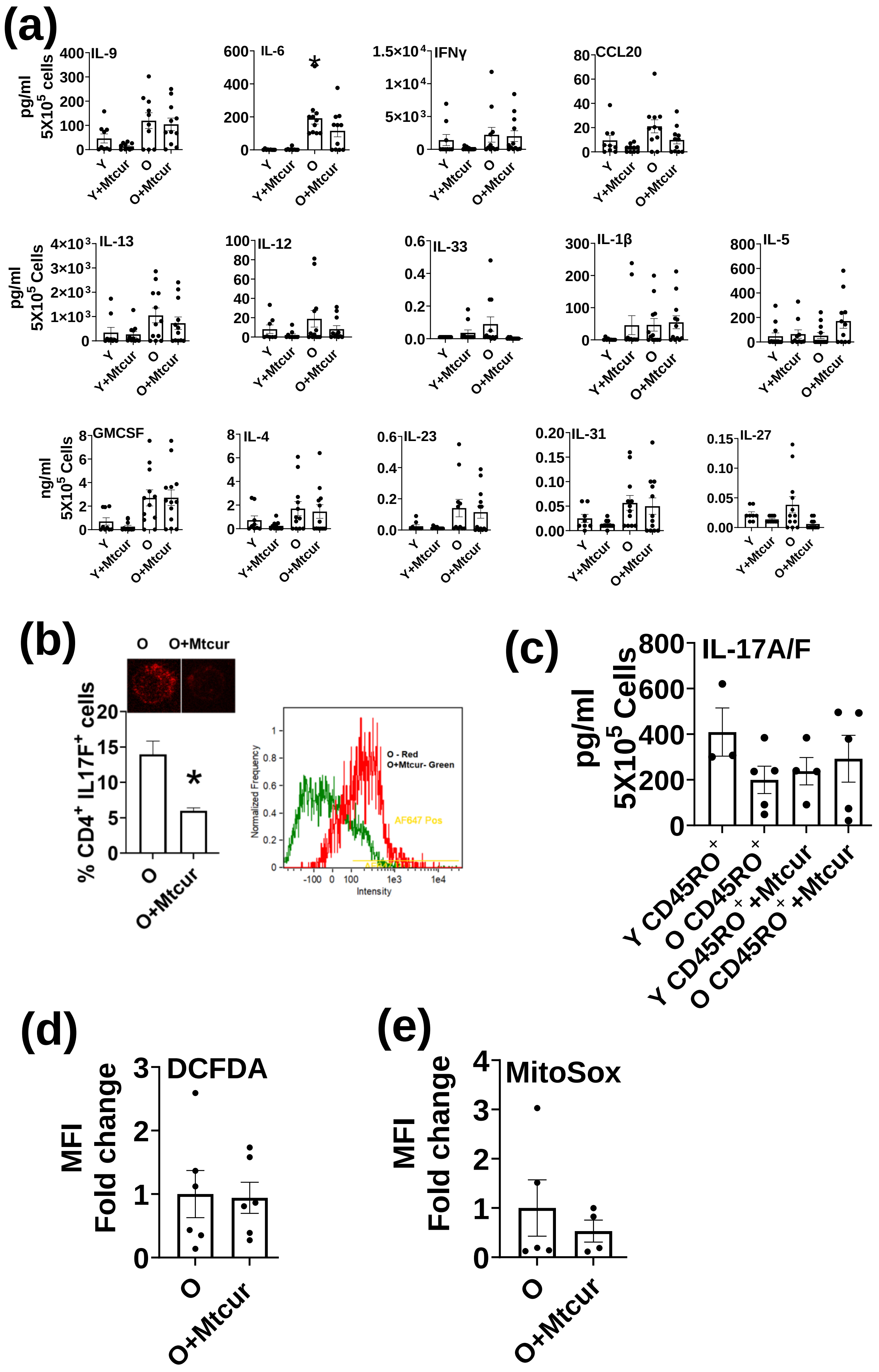

Supplementary Figure 3. Cytokine production in T cells from Y and O adults ± *in vitro* Mtcur treatment **(a)** , Percentage of Th17 effector cells ± Mtcur **(b)** ELISA assay measuring IL17A/F production by memory cells(CD45RO$^+$) **(c)** cellular peroxide **(d)** and mitochondrial superoxide production **(e)** N= 8-12 a, N= 3-6 b-e. * p<0.05 a, c Kruskal Wallis or One way Anova, b,d,e. Wilcoxon test.

Supplementary Figure 4. Distribution of mitoSTAT3 in T cells.

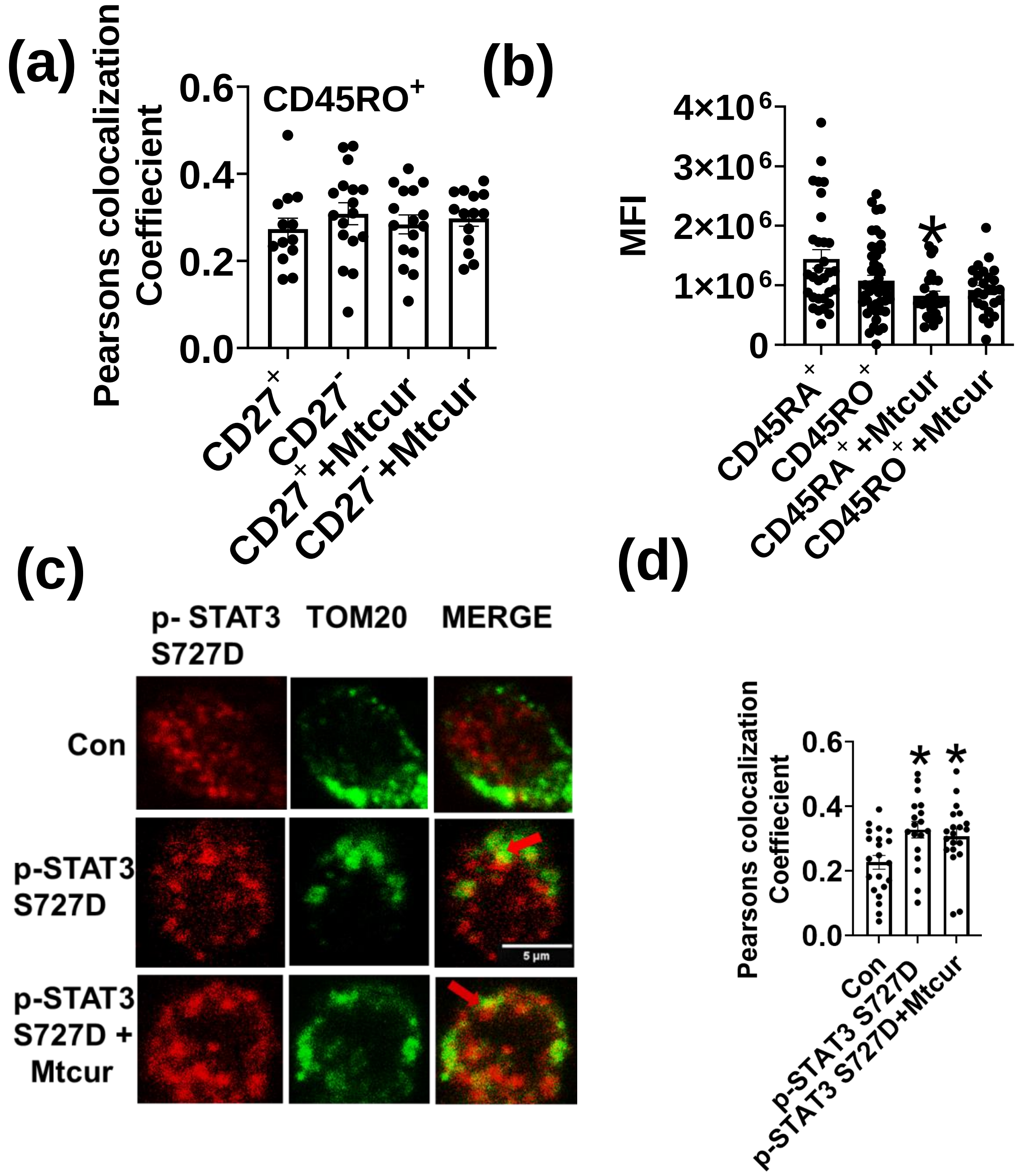

Supplementary Figure 4. Differential distribution of MitoSTAT3 in T cell population. MitoSTAT3 in CD45RO^{+} CD27^{+} and CD45RO^{+} CD27^{-} memory cells from O adults **(a)**, expression of p-STAT3 Ser727 in naïve and memory cells from O adults ± Mtcur **(b)**. Mitochondrial localization of p-STAT3 S727D in Y cells ± Mtcur, representative images **(c)** quantification **(d).** For microscopy, 3-5 fields were imaged. N= 3 a-d, * $p<0.05$ vs. CD27^{+} or CD45RA^{+} or Con, Kruskal Wallis.

Supplementary Figure 5. Production of cytokines in cells expressing p-STAT3 S727D

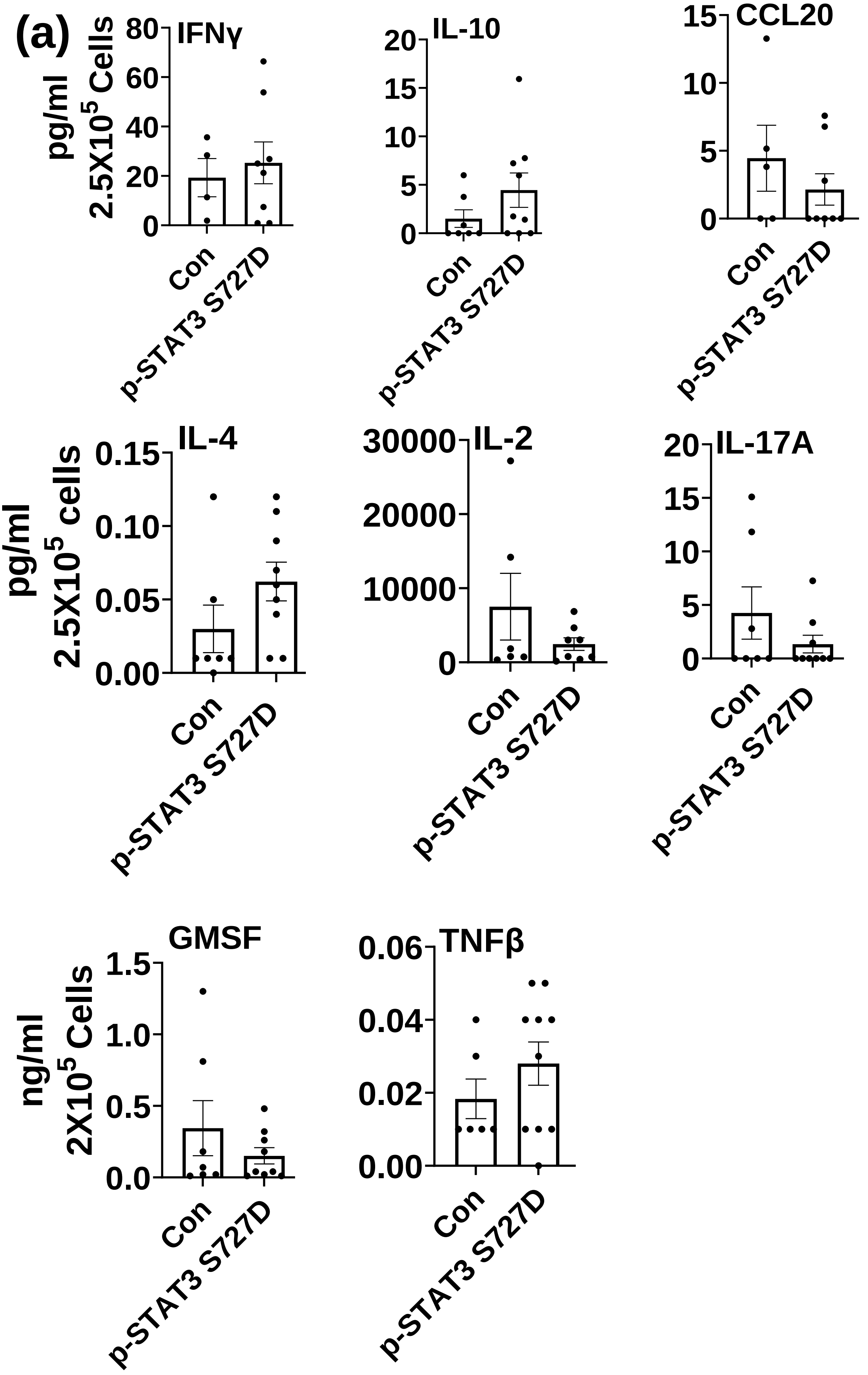

Supplementary Figure 5. Cytokine production in T cells from younger (Y) adults after transfection with either empty vector or p-STAT3 S727D. N= 7-9 **(a)**, Wilcoxon matched-pair signed rank test.
